# Immunoglobulin genes and severity of COVID-19

**DOI:** 10.1007/s00251-024-01341-z

**Published:** 2024-04-11

**Authors:** Daniel Vázquez-Coto, Christine Kimball, Guillermo M. Albaiceta, Laura Amado-Rodríguez, Marta García-Clemente, Juan Gómez, Eliecer Coto, Janardan P. Pandey

**Affiliations:** 1 Instituto de Investigación Sanitaria del Principado de Asturias, ISPA, Oviedo, Spain; 2 Department of Microbiology and Immunology, Medical University of South Carolina, Charleston, SC 29425 USA; 3 Genética Molecular, Hospital Universitario Central Asturias, Oviedo, Spain; 4 Unidad de Cuidados Intensivos Cardiológicos, Hospital Universitario Central Asturias, Oviedo, Spain; 5 Universidad de Oviedo, Oviedo, Spain; 6 CIBER-Enfermedades Respiratorias, Instituto de Salud Carlos III, Madrid, Spain; 7 Instituto Universitario de Oncología del Principado de Asturias, Oviedo, Spain; 8 Neumología, Hospital Universitario Central Asturias, Oviedo, Spain

**Keywords:** GM allotypes, Humoral immunity, SARS-CoV-2, COVID-19, *FCGR2A*, ADCC

## Abstract

There is tremendous interindividual and interracial variability in the outcome of SARS-CoV-2 infection, suggesting the involvement of host genetic factors. Here, we investigated whether IgG allotypes GM (γ marker) 3 and GM 17, genetic markers of IgG1, contributed to the severity of COVID-19. IgG1 plays a pivotal role in response against SARS-CoV-2 infection. We also investigated whether these GM alleles synergistically/epistatically with *IGHG3* and *FCGR2A* alleles—which have been previously implicated in COVID-19—modulated the extent of COVID-19 severity. The study population consisted of 316 COVID-19 patients who needed treatment in the intensive care unit of Hospital Universitario Central de Asturias. All individuals were genotyped for GM 3/17, *IGHG3* hinge length, and *FCGR2A* rs1801274 A/G polymorphisms. Among the 316 critical patients, there were 86 deaths. The risk of death among critical patients was significantly higher in subjects with GM 17 (IgG1) and short hinge length (IgG3). GM 17-carriers were at almost three-fold higher risk of death than non-carriers (*p* < 0.001; OR = 2.86, CI 1.58–5.16). Subjects with short hinge length of IgG3 had a two-fold higher risk of death than those with medium hinge length (*p* = 0.01; OR = 2.16, CI 1.19–3.90). GM 3/3 and *IGHG3* (MM) genotypes were less frequent among death vs. survivors (9% vs 36%, *p* < 0.001) and associated with protective effect (OR = 0.18, 95% CI = 0.08–0.39). This is the first report implicating IgG1 allotypes in COVID-19-spurred death. It needs to be replicated in an independent study population.

**Supplementary Information:**

The online version contains supplementary material available at 10.1007/s00251-024-01341-z.

The clinical spectrum of SARS-CoV-2 infection varies between non-severe or mild symptoms to an exacerbated inflammatory response in the lung that results in severe bilateral pneumonia. Cases with the most severe COVID-19 manifestation require respiratory support in the intensive care unit (ICU) and are at increased risk of thromboembolic events and death. It has long been recognized that IgG subclasses differ significantly in their ability to affect virus neutralization, opsonization of the infected cell, modulation of cytokine production, and mediating effector responses, such as antibody-dependent cell-mediated cytotoxicity (ADCC). ADCC is triggered upon binding of the IgG-constant region to the cellular Fcγ-receptors (FcγR). Antiviral antibody responses, including SARS-CoV-2, are predominantly IgG1 and IgG3. There are hereditary variations within IgG subclasses—called GM (γ marker) allotypes—encoded by immunoglobulin constant heavy G chain (*IGHG*) genes on chromosome 14. Owing to almost complete linkage disequilibrium between particular GM alleles within a race, GM allotypes are inherited in fixed combinations, i.e., haplotypes. Every major racial group is characterized by a unique array of GM haplotypes (Oxelius and Pandey 2013). Although the rsIDs for most single nucleotide polymorphisms (SNPs) characterizing GM allotypes have not been published, rs1071803 appears to code for GM 3/17 (arginine/lysine) on *IGHG1*. There is evidence of differential binding of allotypically different IgG antibodies to the FcγR molecules expressed on effector cells, which could provide a mechanistic explanation for their involvement in ADCC of virally infected cells (Armour et al. 2010; Moraru et al. 2015).

IgG3 subclass is unique in that it varies in its hinge length by 2–4 copies of a 15 amino acid exon-repeat in the *IGHG3* gene. Several studies have demonstrated that increased hinge lengths result in greater IgG3 flexibility that would facilitate its binding to multiple epitopes, resulting in more potent phagocytosis and viral neutralization. IgG3 hinge-length polymorphism has been associated with the severity of infectious diseases, including COVID-19 (López-Martínez et al. 2022a, b). A SNP in the EC2 domain of *FCGR2A*-CD32a (rs1801274, c.497G>A, p.Arg131His) has been implicated in differential IgG-FcγR binding affinity: the ^H^131 isoform has a higher binding affinity to IgG1 compared to the ^R^131 (Bruhns et al. 2009), potentially resulting in increased capacity of binding to IgG immune complexes among individuals with the His/His genotype. *FCGR2A* polymorphisms have been associated with the risk of developing autoimmune diseases and the extent of viral disease, including COVID-19 (Nagelkerke et al. 2019). In some inflammatory conditions (e.g., sarcoidosis), *FCGR2A* variants might serve as prognostic markers of the disease (Typiak et al. 2023).

We previously reported significant associations of *IGHG3* and *FCGR2A* polymorphisms with the risk of developing critical COVID-19 (López-Martínez et al. 2022a, b). Due to the pivotal role of IgG1 in response against SARS-CoV-2, we hypothesized that common functional variants in the *IGHG1* gene—either individually or synergistically with *IGHG3* and *FCGR2A*—might modulate the extent of COVID-19 severity.

The study was approved by the Ethical Research Committee of Asturias, and informed consent was obtained from each patient’s next of kin. All patients were of European ancestry from the region of Asturias (Northern Spain, total population of one million), recruited during the first three pandemic waves (period March 2020 to March 2021). None of the study participants had been vaccinated against SARS-CoV-2. We studied 316 COVID-19 patients (SARS-CoV-2 confirmed by nasopharyngeal PCR) in need of treatment in the ICU of Hospital Universitario Central de Asturias. The pre-existing cardiovascular comorbidities (hypertension, diabetes, dyslipidemia, BMI) from 294 critical patients were obtained from the clinical history at ICU admission. We also studied 136 non-critical COVID-19 patients with symptoms that did not require hospitalization in the ICU. Population controls consisted of 200 sex- and age-matched individuals from the Asturias region; they were recruited before the SARS-CoV-2 pandemic, and no data about the SARS-CoV-2 disease status were available.

DNA was obtained from whole blood leukocytes, and all individuals were genotyped for *IGHG1* rs1071803 C/T (Arg97Lys), *IGHG3* hinge-length, and *FCGR2A* rs1801274 A/G polymorphisms, using previously described procedures (López-Martínez et al. 2022a, b; Pandey et al. 2019).

Demographic and clinical history of patients were obtained at ICU admission. Age was dichotomized as < 65 or ≥ 65 years because this was considered the cutoff age for early vs. late-onset COVID-19 in most studies. Statistical significance of genotype and allele frequency differences between groups was determined by logistic regression, using the R software (version R-4.3.3 for Windows; https://r-project.org). Haplotype frequencies of *IGHG1* rs1071803 and *IGHG3* hinge-length variants were determined online with CubeX (http://apps.biocompute.org.uk/cubex/) (Gaunt et al. 2007).

Main demographic, clinical, and genotype data for the study cohort are presented in Table 1. Of note, among the 316 critical patients, there were 86 deaths. There were no significant differences between controls aged < and ≥ 65 years, so they were grouped together as controls to compare with the patients. Genotype frequencies did not differ significantly between patient age groups.

**Table 1:** Main characteristics of the study cohorts

	Death (*N* = 86)	Critical survive (*N* = 230)	No critical total (*N* = 136)	Critical total (*N* = 316)	Controls (*N* = 200)
Male	68 (79%)	157 (68%)	102 (75%)	225 (71%)	102 (51%)
Age range	38–85	21–85	30–89	21–85	35–85
Age < 65 years	19 (22%)	121 (53%)	80 (59%)	201 (64%)	90 (45%)
	***IGHG1*-rs1071803, GM 17 (lysine) vs. GM 3 (arginine)**				
GM 17/17	22 (26%)	42 (18%)	18 (13%)	64 (20%)	25 (13%)
GM 3/17	47 (55%)	93 (40%)	68 (50%)	140 (44%)	82 (41%)
GM 3/3	17 (20%)	95 (42%)	50 (37%)	112 (36%)	93 (46%)
Allele GM 17	0.53	0.38	0.38	0.42	0.33
	*p* < 0.001 OR = 2.86 (1.58–5.16)			*p* = 0.01 OR = 1.58 (1.10–2.27)	
	***IGHG3*, hinge length, *M* = 4-repeats, *S* = 3-repeats**				
MM	62 (72%)	195 (85%)	106 (78%)	257 (81%)	176 (88%)
MS	23	32	28	55	22
SS	1	3	2	4	2
S	0.15	0.15	0.12	0.15	0.07
	*p* = 0.01 OR = 2.16 (1.19–3.90)			*p* = 0.04 OR = 1.68 (1.01–2.81)	
	***FCGR2A*, rs1801274, c.497G > A, p. Arg131His**				
AA	11 (13%)	51 (22%)	38 (28%)	62 (20%)	45 (22%)
AG	49 (57%)	126 (55%)	55 (40%)	175 (55%)	101 (51%)
GG	26 (30%)	53 (23%)	43 (32%)	79 (25%)	54 (27%)
G (Arg)	0.59	0.50	0.48	0.53	0.52
	*p* = 0.06			n.s	

The risk of death among critical patients was significantly higher in subjects with GM 17 (IgG1) and short hinge length (IgG3). GM 17-carriers were at almost three-fold higher risk of death than non-carriers (*p* < 0.001; OR = 2.86, CI 1.58–5.16). Subjects with short hinge length of IgG3 had a two-fold higher risk of death than those with medium hinge length (*p* = 0.01; OR = 2.16, CI 1.19–3.90). There was a non-significant trend toward increased risk of death among *FCGR2A* rs1801274 G (^R^131) carriers (*p* = 0.06). There were no significant allele and genotype differences between critical and non-critical patients for the three polymorphisms. Compared to controls, the *IGHG1*-GM 17 and *IGHG3*-S variants were associated with an increased risk of critical COVID-19 (Table 1).

The *IGHG1* rs1071803 and *IGHG3* hinge-length variants were in low linkage disequilibrium in our population (controls, D = 0.125, *r*^2^ = 0.0022), with GM 17-M as the most common haplotype. The haplotype frequencies in various study groups are given in the supplementary Table 1. The *IGHG1-IGHG3* risk genotype combinations are given in supplementary Table 2. *IGHG1* (GM 3/3) and *IGHG3* (MM) genotypes were less frequent among death vs. survivors (9% vs 36%, *p* < 0.001) and associated with protective effect (OR = 0.18, 95% CI = 0.08–0.39, Fig. 1). Taking IgG1+ IgG3 non-risk genotypes as reference, the OR for the IgG1+IgG3 risk genotypes was higher than that of IgG1 or IgG3 separately, suggesting an epistatic interaction between the two loci in the risk of death (supplementary Table 2).

**Fig. 1:**
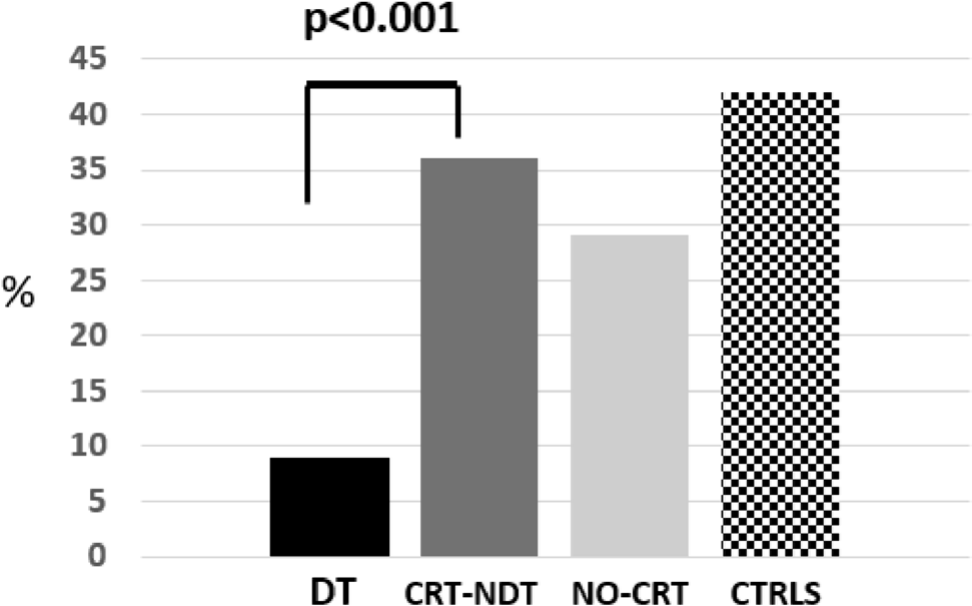
Frequency of carriers of the non-risk genotypes *IGHG1* (GM 3/3) and *IGHG3* (MM) in death (DT), critical survivors (CTR-NDT), non-critical patients (NO-CRT), and population controls (CTRLS). The two genotypes were significantly less common among the death vs survivors (9% vs 36%, *p* < 0.001) with a protective effect (OR = 0.18, 95% CI = 0.08–0.39)

The results of this investigation clearly show that among critically ill COVID-19 patients, those expressing the GM 17 allotype of IgG1 are at much greater risk of death, whereas those expressing the alternate genotype, GM 3/3, are protected from COVID-19-spurred death. There is precedence for GM 17 association with other viral infections. For instance, GM 17 is associated with symptomatic cytomegalovirus infection (Di Bona et al. 2018). In hepatitis C virus infection, subjects with GM 17 have been shown to be at higher risk of infection while those with the alternate GM 3 allele were relatively protected (Vejbaesya et al. 2004).

Although the exact mechanisms underlying the association of GM 17 with COVID-19-spurred death—and GM 3 with relative protection—are not understood, one can speculate based on the demonstrated role of IgG subclasses in antibody responses to SARS-CoV-2 and the influence of GM allotypes on IgG subclass levels and on Fc-mediated effector functions. Subjects positive for GM 17 (expressed on IgG1) are most likely also positive for GM 21 (expressed on IgG3), as the two alleles are in absolute linkage disequilibrium in European populations (Oxelius and Pandey 2013). GM 21 has been shown to be associated with low IgG3 levels by many studies (Grubb 1995). IgG3 plays a pivotal role in protection from infectious pathogens in general, including SARS-CoV-2. A recent study reported up to 50-fold increased neutralization potency of an anti-SARS-CoV-2 monoclonal IgG3 antibody, compared to antibodies of the other three IgG subclasses with identical antigen-binding site (Kallolimath et al. 2021). The heightened potency of IgG3 antibodies has been attributed to its long hinge region. In addition to this possibility, it has been postulated that GM allotypes expressed on IgG3 could influence the potency of anti-SARS-CoV-2 IgG3 antibodies (Pandey 2022). The highly potent IgG3 antibody expressed the GM 5 allotype, which is in absolute linkage disequilibrium with GM 3, shown to be associated with relative protection from COVID-19-spurred death in the current study. The protective effect of GM 3 can also be explained by its possible interaction with FcγRIIIa on NK cells, resulting in ADCC of SARS-CoV-2-infected cells. This mechanism has been documented for HSV1-infected cells (Moraru et al. 2015).

We have not measured anti-SARS-CoV-2 IgG3 antibody levels in this study population, but based on the established association of GM 21 with low IgG3 levels (Grubb 1995), it is reasonable to assume that GM 17 (21) positive subjects, who were at high risk of death, probably induced low levels of virus-specific IgG3 antibodies, making them less immunocompetent and thus susceptible to SARS-CoV-2-spurred death.

The association of the *IGHG3* hinge length polymorphisms with the risk of critical disease and death has been previously reported (López-Martínez et al. 2022a, b). We previously reported a significantly increased frequency of the rs1801274 G (131^Arg^) among COVID-19 deaths (López-Martínez et al. 2022a, b). In this study, the G allele was non-significantly more common among deaths vs. survivors (*p* = 0.06). We did not find a significant interactive effect of IgG1, IgG3, and *FCGR2A* variants on survival (data not shown). In a small study, GM 23 (expressed on IgG2) was marginally associated with symptomatic SARS-CoV-2 infection (Ligotti et al. 2022).

In addition to advanced age and male sex, hypertension, diabetes, dyslipidemia, and obesity have been associated with increased risk of critical COVID-19. We did not find a significant difference in the risk genotypes between the presence/absence of the cardiovascular traits (supplementary Table 3). The risk of death was significantly associated with the presence of IgG1-GM17 and *IGHG3*-S after multiple logistic regression corrections for hypertension, diabetes, dyslipidemia, BMI ≥ 30, sex, and age.

To our knowledge, this is the first report implicating IgG1 allotypes in COVID-19-spurred death. It needs to be replicated in a larger and independent study population. Also, since every major race is characterized by a unique array of GM haplotypes (Oxelius and Pandey 2013), a multiethnic cohort needs to be investigated to determine whether the findings presented here can be generalized.

### Supplementary Information

Below is the link to the electronic supplementary material.

Supplementary file1 (DOCX 20 KB)

Supplementary file2 (DOCX 23 KB)

Supplementary file3 (DOCX 14 KB)

## Data Availability

The data generated in this study will be available from the corresponding authors upon reasonable request.
