# Supplementary material for: Immunoglobulin genes and severity of COVID-19
Source: Immunogenetics. 2024 Apr 11;76(3):213–7. doi: 10.1007/s00251-024-01341-z (PMC11087305; doi:10.1007/s00251-024-01341-z)
Supplement: Supplementary file 1 — Supplementary file1 (DOCX 20 KB) [file 251_2024_1341_MOESM1_ESM.docx]

Supplementary Table 1 GHG1 (GM 17)-IGHG3 (M,S) haplotype frequencies

|  | DEATH  N=172 | CRIT SURVIVORS  N=460 | NON-CRITICAL  N=272 | CONTROLS  N=400 |
| --- | --- | --- | --- | --- |
| GM 17-M | 0.52 | 0.36 | 0.33 | 0.30 |
| GM 17-S | 0.02 | 0.02 | 0.06 | 0.03 |
| GM 3-M | 0.34 | 0.56 | 0.56 | 0.63 |
| GM 3-S | 0.13 | 0.06 | 0.06 | 0.04 |
