# Supplementary material for: Immunoglobulin genes and severity of COVID-19
Source: Immunogenetics. 2024 Apr 11;76(3):213–7. doi: 10.1007/s00251-024-01341-z (PMC11087305; doi:10.1007/s00251-024-01341-z)
Supplement: Supplementary file 2 — Supplementary file2 (DOCX 23 KB) [file 251_2024_1341_MOESM2_ESM.docx]

Supplementary Table 2. IGHG1-IGHG3 risk genotype combination.

IGHG1-risk=presence of GM 17,

IGHG3-risk=presence of short length (S),

FCGR2A-risk=presence of rs1801274 G

| IGHG1  risk | IGHG3  risk | Death  N=86 | Crit  surv  N=230 | **OR** | TOTAL CRIT  N=316 | CONTROLS  N=200 | **OR** | No critical  N=136 |
| --- | --- | --- | --- | --- | --- | --- | --- | --- |
| NO | NO | 8 (9%) | 84 (36%) | **REF** | 92 (29%) | 83 (42%) | **REF** | 40 (29%) |
| NO | YES | 9 (10%) | 14 (6%) | **6.75** | 23 (7%) | 10 (5%) | **2.08** | 10 (7%) |
| YES | NO | 54 (63%) | 111 (49%) | **5.11** | 165 (52%) | 93 (47%) | **1.60** | 66 (49%) |
| YES | YES | 15 (17%) | 21 (9%) | **7.5** | 36 (11%) | 14 (7%) | **2.32** | 20 (15%) |

| IGHG1  risk | FCGR2A  risk | Death  N=86 | Crit surv  N=230 | **OR** | TOTAL CRIT  N=316 | CONTROLS  N=200 | **OR** | No critical  N=136 |
| --- | --- | --- | --- | --- | --- | --- | --- | --- |
| NO | NO | 2 (9%) | 21 (36%) | **REF** | 23 (7%) | 23 (12%) | **REF** | 15 (11%) |
| NO | YES | 15 (10%) | 74 (6%) | **2.12** | 89 (28%) | 70 (35%) | **1.27** | 35 (26%) |
| YES | NO | 9 (63%) | 28 (49%) | **2.41** | 37 (12%) | 22 (11%) | **2.30** | 23 (17%) |
| YES | YES | 60 (17%) | 107 (9%) | **4.20** | 167 (53%) | 85 (42%) | **1.96** | 63 (46%) |
