# Supplementary material for: Immunoglobulin genes and severity of COVID-19
Source: Immunogenetics. 2024 Apr 11;76(3):213–7. doi: 10.1007/s00251-024-01341-z (PMC11087305; doi:10.1007/s00251-024-01341-z)
Supplement: Supplementary file 3 — Supplementary file3 (DOCX 14 KB) [file 251_2024_1341_MOESM3_ESM.docx]

|  | IGHG1 risk  (GM17+) | IGHG3 risk  (S+) | FCGR2A risk  (G+) |
| --- | --- | --- | --- |
| Hypertension yes (165) | 110 (67%) | 28 (17%) | 136 (82%) |
| Hypertension no (129) | 78 (61%) | 27 (21%) | 100 (78%) |
|  | p=0.28 | p=0.20 | p=0.38 |
| Diabetes yes (67) | 46 (69%) | 9 (13%) | 53 (80%) |
| Diabetes no (227) | 142 (63%) | 46 (20%) | 183 (81%) |
|  | p=0.36 | p=0.21 | p=0.78 |
| Dyslipaemia yes (67) | 90 (64%) | 27 (19%) | 110 (82%) |
| Dyslipaemia no (227) | 98 (64%) | 28 (18%) | 126 (78%) |
|  | p=0.97 | p=0.85 | p=0.35 |
| BMI ≥30 yes (145) | 91 (63%) | 24 (17%) | 102 (80%) |
| BMI ≥30 no (127) | 83 (65%) | 28 (21%) | 118 (81%) |
|  | p=0.66 | p=0.25 | p=0.82 |

Supplementary Table 3. Distribution of the three gene variants in critical patients (N=294) according to the cardio-vascular risk factors
